# What makes health systems resilient? A qualitative analysis of the perspectives of Swiss NGOs

**DOI:** 10.1186/s12992-022-00848-y

**Published:** 2022-05-26

**Authors:** Pauline Yongeun Grimm, Kaspar Wyss

**Affiliations:** 1 Swiss Centre for International Health, Swiss Tropical and Public Health Institute, Basel, Switzerland; 2 University of Basel, Basel, Switzerland

**Keywords:** Health system resilience, Complex shocks, Qualitative research, NGOs

## Abstract

**Background:**

Resilience has become relevant than ever before with the advent of increasing and intensifying shocks on the health system and its amplified effects due to globalization. Using the example of non-state actors based in Switzerland, the aim of this study is to explore how and to what extent NGOs with an interest in global health have dealt with unexpected shocks on the health systems of their partner countries and to reflect on the practical implications of resilience for the multiple actors involved. Consequently, this paper analyses the key attributes of resilience that targeted investments may influence, and the different roles key stakeholders may assume to build resilience.

**Methods:**

This is a descriptive and exploratory qualitative study analysing the perspectives on health system resilience of Swiss-based NGOs through 20 in-depth interviews. Analysis proceeded using a data-driven thematic analysis closely following the framework method. An analytical framework was developed and applied systematically resulting in a complete framework matrix. The results are categorised into the expected role of the governments, the role of the NGOs, and practical future steps for building health system resilience.

**Results:**

The following four key ‘foundations of resilience’ were found to be dominant for unleashing greater resilience attributes regardless of the nature of shocks: ‘realigned relationships,’ ‘foresight,’ ‘motivation,’ and ‘emergency preparedness.’ The attribute to ‘integrate’ was shown to be one of the most crucial characteristics of resilience expected of the national governments from the NGOs, which points to the heightened role of governance. Meanwhile, as a key stakeholder group that is becoming inevitably more powerful in international development cooperation and global health governance, non-state actors namely the NGOs saw themselves in a unique position to facilitate knowledge exchange and to support long-term adaptations of innovative solutions that are increasing in demand. The strongest determinant of resilience in the health system was the degree of investments made for building long-term infrastructures and human resource development which are well-functioning prior to any potential crisis.

**Conclusions:**

Health system resilience is a collective endeavour and a result of many stakeholders’ consistent and targeted investments. These investments open up new opportunities to seek innovative solutions and to keep diverse actors in global health accountable. The experiences and perspectives of Swiss NGOs in this article highlight the vital role NGOs may play in building resilient health systems in their partner countries. Specifically, strong governance, a bi-directional knowledge exchange, and the focus on leveraging science for impact can draw greater potential of resilience in the health systems. Governments and the NGOs have unique points of contribution in this journey towards resilience and bear the responsibility to support governments to prioritise investing in the key ‘foundations of resilience’ in order to activate greater attributes of resilience. Resilience building will not only prepare countries for future shocks but bridge the disparate health and development agenda in order to better address the nexus between humanitarian aid and development cooperation.

**Supplementary Information:**

The online version contains supplementary material available at 10.1186/s12992-022-00848-y.

## Background

Since the 2014–15 Ebola epidemic in West Africa, a deluge of literature has been published to elucidate the concept within the health sector, as traditionally the term had been discussed mainly in the engineering sciences, ecology and developmental psychology [1–4]. Health system resilience is by and large described as the capacity to prepare for and effectively respond to crises whilst retaining core health system functions [5].

Varying conceptual frameworks help break down the complex nature of health system resilience. One of the first frameworks developed was that of Kruk et al. in the aftermath of the Ebola crisis, responding to the growing demands of multilateral organisations to illustrate the key characteristics of a resilient health system and a proposed resilience index to measure resilience [6]. Blanchet et al. suggested a new model of resilience as an underlying management and governance capacity to absorb, adapt and transform itself in case of a shock [7]. Gilson et al. introduced the idea of ‘everyday resilience,’ highlighting the strategies and capacities that are required to address both chronic stressors as well as acute shocks in the health system [8–10]. Grimm et al. have synthesized empirical studies from low- and middle- income countries to build on Kruk et al’s five characteristics of resilience and to identify five additional characteristics that serve as foundations that may be prioritized in resource-constrained settings to activate system-level resilience [11]. The five foundations of resilience are ‘realigned relationships,’ ‘foresight,’ ‘motivation,’ ‘changed management,’ and ‘emergency preparedness,’ which have been identified as foundational for health systems to unlock the following attributes of resilience described by Kruk et al.: ‘aware,’ ‘diverse,’ self-regulating,’ ‘integrated,’ and ‘adaptive’ [5, 11].

A critical consideration for the discourses of health system resilience that has not been widely discussed is the state’s level of fragility, which may undermine efforts to build resilience in the first place [12]. As many NGOs that part took in this study operate in fragile, weak or failed states, this paper will give full acknowledgement to the underlying contextual complexities that could affect one’s journey towards resilience.

As the world has wrestled with a pandemic that has intimately affected the course of our daily lives, the level of extreme weather events, natural disasters, conflicts, and economic recession are amplifying existing inequalities. The United Nations Office for Disaster Risk Reductions (UNDRR) reported that there has been a two-fold increase in climate-related disasters in the past twenty years compared with the previous twenty years, with evidence of climate change increasing the frequency and intensity of extreme weather events in the future [13]. The International Rescue Committee (IRC) has released its 2021 Emergency Watchlist of humanitarian crises that are expected to deteriorate over the coming years, exacerbated by the triple threat of conflict, climate change and COVID-19 [14]. Research on health system resilience is highly relevant in the twenty-first century in such a context where an interlinked global economy, porous borders and easy influx of people, goods, and services imply pathogens to travel at a much faster pace than control measures can be put in place. The effects of globalization have reverberated through the world’s undergoing the COVID-19 pandemic and have outpoured publications on the topic of resilience in health systems around the world.

In response to the increasing frequency and intensity of these catastrophic events, various international organisations have released consolidated evidence and guidance for policy makers and relevant stakeholders to incorporate resilience into policy and planning. For example, the World Bank’s latest report outlines five pillars of resilient health systems and offers priority areas for government actions, drawing upon lessons from disaster risk and emergency management practices [15]. World Health Organisation (WHO)‘s Regional Office for Europe has also put forward a new policy brief, outlining concepts and strategies to strengthen health system resilience and providing proposed indicators to assess resilience by topic area [16]. Despite such promising momentum, the link between applied research and implementation remain at infant stages and gaps exist in streamlining and contextualizing this gargantuan list of recommendations for practitioners.

This paper addresses this nexus by adding to the empirical evidence of health system resilience through analysing the perspectives of NGOs working in the field of global health being regularly exposed to shocks and at the same time acting in the forefronts of health service delivery and health systems operations. As a key stakeholder group in international development cooperation and global health governance, non-state actors namely NGOs have responsibilities to protect and work alongside the national governments in times of crises and to offer appropriate support in the areas where they cause no further harm.

### Objectives

Using the example of non-state actors based in Switzerland, the aim of this study is to examine how and to what extent NGOs have dealt with unexpected shocks in the health systems of their partner countries and to reflect on the practical implications of resilience for the multiple actors involved. This paper analyses the key attributes of resilience that targeted investments may influence, and explores the different roles key stakeholders may assume to build resilience.

## Methods

### Study design

This is a descriptive and exploratory qualitative study analysing the practical experience and perspectives of health system resilience through 20 in-depth interviews of Swiss-based NGOs conducted between April and June of 2021.

### Study participants

The participants were purposively selected through a snowball sampling of relevant referrals from Medicus Mundi Switzerland [17], a network comprised of Swiss organisations active in international health cooperation. Organisations with a global outreach and an active ongoing health portfolio in low- and middle- income countries were included. Albeit the heterogeneity of the organisation’s target groups and approaches to programmes, the 20 interviews nevertheless represent a single stakeholder group and therefore has shown to be sufficient to reach data saturation [18].

### Data collection

The interviews lasted around 1 hour respectively and were conducted by the first author (PYG) in English. There have been no prior exchanges between the participants and the data collector and the interviews were held on zoom due to the pandemic restrictions. A semi-structured interview guide was developed to explore the following key topics: 1) background of the participant’s organisation; 2) experience of health system shocks in partner countries; 3) perceptions of health system resilience and its dimensions; 4) self-assessment; 5) preparedness for future shocks. All interviewees were prompted on their views on key resilience attributes based on the respective shocks experienced. See appendix 1 for the full interview guide. All interviews have been audio-recorded following informed consent and thereafter transcribed verbatim. Independent of the number of interviews to approximate data saturation, an iterative approach to data collection and analysis confirmed that data saturation was indeed reached as a result.

### Data analysis

**Fig. 1:**
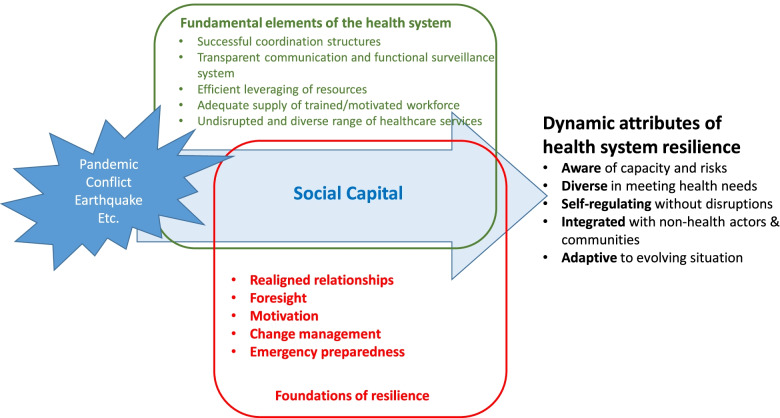
Grimm et al’s health system resilience framework [11]

Analysis proceeded using a data-driven thematic analysis, closely following the steps of the framework method [19]. Verbatim transcriptions were reviewed for familiarization and verified for accuracy. Following the development of an initial codebook inspired by the ten themes previously developed by the author’s team [11], all transcripts were coded line-by-line using MAXQDA 2018. A hybrid of deductive and inductive approaches provided for a broader structure of the categories and flexibility of codes from the open coding process. The codebook, themes and categories initially developed by the first author (PYG) were reviewed and validated by the second author (KW). Any differences in interpretation were resolved through internal discussions. As depicted in Fig. 1, Grimm et al’s conceptual framework was used to develop the analytical framework. The framework was then applied systematically to chart the relevant summary of the transcript with direct quotations structured around 14 categories and 37 codes, resulting in a complete framework matrix. This process enabled comparisons and contrasts of key emergent themes between the participants, which later generated key results describing the prominent dimensions of health system resilience and the key roles expected of stakeholders to build resilience in the health systems.

## Results

Twelve female and eight male interviewees participated. The majority of the participants were either managing directors or project managers of Swiss NGOs having operations in low- and middle- income countries. Two of the interviewees were based in the partner country as project managers, and the remaining participants were based in Switzerland having close and regular contact with their respective in-country partners. Most organisations have ongoing project engagements in Africa, whilst a few of them oversee projects in Asia, Middle East, Eastern Europe, and South America. The primary target groups of the key informants’ organisations are summarised in Table 1.

**Table 1:** Overview of key informants’ organisational primary target group(s)

Primary target group(s) of the NGO^a^	Total
Women and pregnant mothers (4)	4
Communities (7)	7
Health workers, veterinarians, midwives (12)	12
Youth and children (5)	5
People living with HIV/AIDS and LGBTQ (4)	4
Persons with disabilities (2)	2
Total priority target groups	34

^a^multiple responses were possible

The results section presents key findings elucidating the practical experience and perspectives of Swiss-based NGOs supporting health systems in low- and middle- income countries. The results are categorised into the role of the governments, the role of the NGOs, and practical future steps for building health system resilience.

### Governments: governance and responsibility

#### Health systems with strong ‘foundations of resilience’ may unleash greater attributes of resilience

**Fig. 2:**
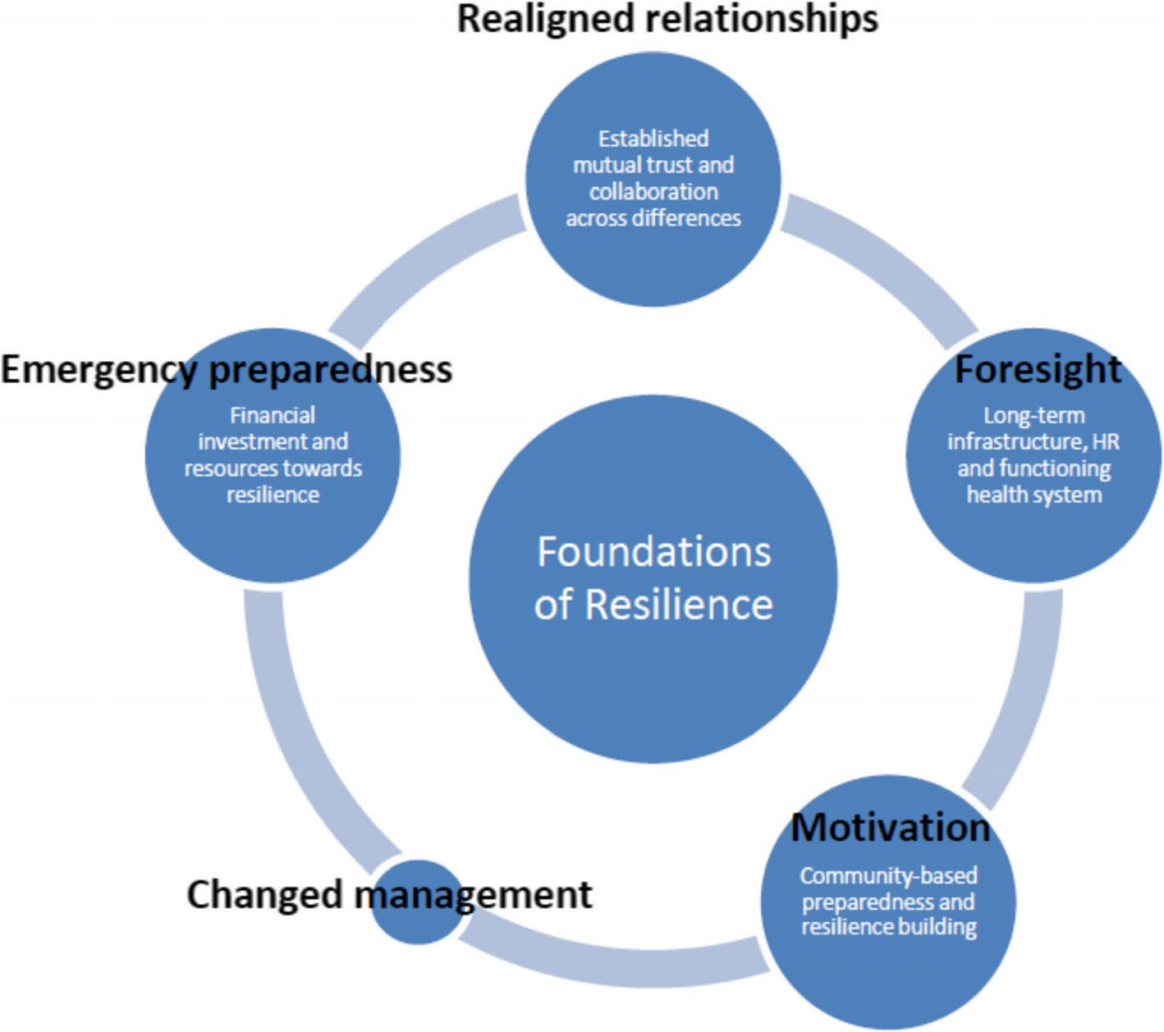
Dominant foundations of resilience among NGO respondents

The participants overall had a solid understanding of health system resilience based on their experience managing programmes and projects in low- and middle- income countries. When asked of the most critical component of resilience, the responses fit into one of the foundations of resilience that serve as inputs into the health system that would activate resilience attributes described in Grimm et al’s framework [11]. As depicted in Fig. 2, among the five foundations, four were considered dominant for unleashing resilience attributes. ‘Change management,’ however, did not emerge as a key foundation from the interviews.

The first foundational element is ‘realigned relationships,’ which was seen to provide a strong basis of resilience at the system level. Establishing trust and collaborations across differences appeared in particular to offer a source of strength against all kinds of external shocks.

*"But, these (external shocks) are not the biggest headaches as long as you have a good team and partnerships that are build on trust." (I03)*

A participant stressed the importance of investing in the initial networking and building of connections as they form the foundation of solid working relationships that can overcome and counteract future shocks. These good working relationships on the ground were what enabled projects to proceed with minimal interference albeit wider contextual disruptions. The answer lies not in a hypothetical system, but in the people that own and carry out the vision of the projects.

*"If you are really engaged with the technocrats on the ground who are still working in the health system, you can still achieve lots of your goals. So, I think the investments especially in the beginning, the investments into the networking and the building of the relationships are key to overcoming and counteracting those shocks which anyway you cannot influence. If a president dies, he dies, and of course there is a state of emergency and so on, but as long as you have good relationships on the ground, people are still willing to continue with the project because they believe in it, having a strong ownership." (I06)*

Building trust, however, does not spring up organically; rather, it entails longstanding collaboration, a cadre of trained staff, an established organisational structure and most importantly a shared vision that stems from a collective sense of ownership.

*"In order to establish the trust, you also need some structure, trained staff, you need a mindset to be able to go there and do that." (I15)*

The second foundational element ‘foresight’ helps to see the importance of investing in long-term functional infrastructure, prioritising in human resource readiness, and focusing on overall health system strengthening. One participant pointed out that a functioning health system, which is not guaranteed in all countries, should take precedence over resilience building.

More important than having a specific disaster preparedness plan is obviously to strengthen the overall health system. Several participants underscored that a preventive approach to resilience is especially necessary in low- and middle- income settings, as the building of infrastructure, equipment, well-trained health workers and responsive communities take intentional investments to cultivate.

*"to make it more resilient, I think it is less important to have a specific disaster preparedness, but rather to strengthen the health system as such.. It's not about once you have a crisis, then you need to react. You need to really strengthen the system before. Indeed a system with a sufficient number of infrastructure, equipment, medication, well-trained health workers, and community which is interacting well, is quite resilient." (I08)*

The third foundational element ‘motivation’ when instilled at the community level builds community-based preparedness and resilience, a precondition for enabling health system resilience. In resource-constrained settings, community resilience can be one of the best preventive strategies for countries to build health system resilience. One respondent explicitly depicted communities as the “drivers of the health system,” an indispensable gateway to project sustainability.

*“Not only the perception of integration, of participation, on feedback mechanisms, but also as the driver of the health system. Communities are part of our common approaches and most of our projects acknowledge sustainability through communities." (I04)*

In the context of failed or fragile states, strong community structures may serve as the sole source of continuous development and fill vacuums created by the lack of state governance. This was apparent in the case of one of the respondents working in Afghanistan where the solidarity from the communities abridged decision-making structures and facilitated resilience.

*“Sometimes you could not work with the government, in the case of Taliban. Then, the development agency tries to build up the community structures and then strengthening them. They are still there and always there. Especially in times of crises they can easily be activated and there is a lot of solidarity and short decision-making structures and agile. This is a big factor when it comes to resilience of a community and of supporting the health system." (I06)*

The fourth foundational element ‘emergency preparedness’ was best exhibited through financial investments and resource preparedness, vital prerequisites to activating resilience. Many highlighted that resources are the bedrock of any development work including that of resilience building. The consequence of financial gaps in fragile states that resulted in shortages of all basic needs were proven detrimental, exacerbating vulnerability to future shocks.

*"If we look into some of the things they've done well, like investments in the adequate health service package in the health facilities, it always comes with some resources obviously. Finance gaps are critical and we see this in countries like Yemen, Somalia, Afghanistan and Iraq, where the gaps in terms of all basic needs, in terms of health are so massive" (I04)*

There is always a trade-off, however, in investment decisions, which also applies in the case of investing in resilience. A respondent recommended that countries set aside a portion of their GDP dedicated to resilience, whether it takes the form of a social insurance or improved health coverage. Financial investments towards emergency preparedness and resilience building are undoubtedly a precondition for a strong foundation of resilience.

*"So, in my point of view, it is a matter of money.. as there is always a trade-off in decisions. For instance, do we do less cancer treatment and invest in resilience?" (I01)*

*"(For resilience building I would advise the government) Make sure you have enough financial resources. 20% of your GDP? You need financial resources. The work to increase these resources and to decrease individual risks through social insurance system. Work towards universal health coverage and health insurance." (I08)*

#### Key resilience attributes expected of national governments: ‘integrate,’ ‘adaptive,’ ‘diverse’

**Fig. 3:**
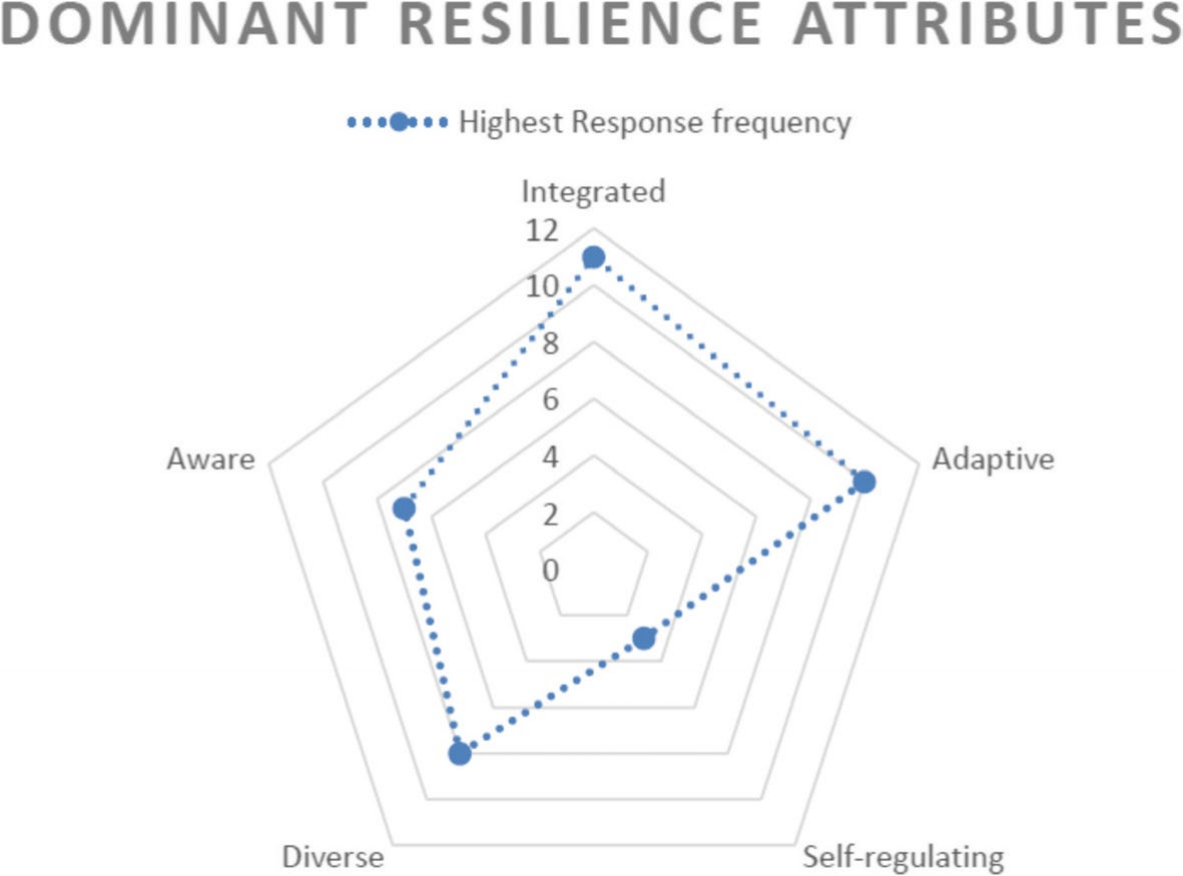
Dominant resilience attributes

The attribute to ‘integrate’ was shown to be one of the most significant characteristics of resilience expected of the national governments from the NGOs, followed by the attributes to be ‘adaptive’ and ‘diverse,’ as depicted in Fig. 3. The figure visualises the frequency of responses by the participants when asked about the most prominent feature of resilience expected of governments.

Most of the participants in one way or another emphasized how important it is to have a solid governance system and to have a government that exemplifies responsibility in responding to health system shocks. Governments were ultimately seen as duty bearers, and the sustainability of any project requires good governance to plan, invest, and anticipate future shocks.

*"(to build resilience you need) good governance. Governments are the duty bearers and you can have shiny health infrastructure and train staff for a period of time but then if it's not sustained with good budget planning, good investment, good governance, then everything can fall apart quickly." (I20)*

The NGOs expected the governments to assume a stronger coordination role to minimise duplication amongst external and internal partners, and to provide guidance based on the needs of the population. A respondent echoed that a badly equipped primary health care system often stems from an underlying fragility of governance, more so than from dilapidated infrastructures or ill-trained staff.

*"What I often see what is lacking is a really strong coordination role of the government to make the best out of what is coming from the inside and outside. Often there is a lack of guidance to be honest. We have still issues where there are two organisations doing exactly the same work in the same region which is a waste of money. More guidance would also help in making the system more resilient." (I06)*

Though many partner countries may not have foresight and long-term planning as strengths, they exhibited exceptional skills to be ‘adaptive,’ which was proven more vital in fragile contexts where the shocks are multiple and continuous. One participant described this ‘adaptive’ attribute as an attitude to change, switch gears and seek new ways to deal with the evolving situation when thorough planning is not possible.

*"I think the attitude to be able to change, switch gears and to find new ways and to be creative somehow and to deal with situations where you can't plan everything, this resilience, that's a resort where i've learnt and still learn from Uganda." (I03)*

Another respondent underscored that this attitude to be adaptive and flexible was a clear indication of resilience shown by the Haitians responding to the devastating earthquake in 2010. There was clear self-sufficiency to intervene even before any external help had access to the area. This improvisation is a clear indication of the ‘adaptive’ attribute of resilience.

*"A part of resilience of the Haitian people is, they for me, they are the best at improvisation. They are very bad at preparation and foreseeing. They are very bad at anticipation. I remember when we had the floodings before the earthquake, immediately before and after the collapsed bridges, they have built up kiosks and you could get transports through the rivers. That's the way we could transport from one side to the other side. Thanks to this improvation. It was the same after the earthquake. Even without the help of external organisations, privately they have built up streets. Well, this is a positive side of resilience." (I05)*

The third most prominent resilience attribute expected of the governments was to be ‘diverse,’ allowing care to extend to new and diverse needs arising from the crises. Endemic health problems, such as cholera and measles, forced governments to make difficult decisions between conflicting priorities. If the shock was substantial, the already scarce resources were redirected to dealing with the immediate crisis whilst leading to major disruptions in routine care.

*"If you take the case of DRC, it's a clear example. Very recently we had Ebola now. But, even before we had other Ebola outbreaks. You could have seen that COVID was not seen as an issue because they had at the same time, cholera, Ebola, measles and other outbreaks. So for them, in terms of prioritisation, you will see that is difficult. (I04)*

*"What we noticed but our partners is that already scarce resources that were there are redirected then to COVID-19 sensitization, PPE and taken away by budget that you actually have for other purposes. This is something all partners experienced and noticed." (I15)*

Health needs arising specifically due to the crisis, such as mental health issues, are often neglected and undervalued. In a resource-constrained setting, life-saving and treatment-based approaches crowd out equally key services such as trauma care and counselling services.

*"I think the mental health systems are undervalued and understaffed. As global North, we are not prepared for the fall-out of the pandemic from a mental health aspect and when I think about that it's such a taboo even where it's much more available versus in low-income countries, it's very much relying on the informal networks. And that's something we are worried about. Take India as an example. Post traumatic stress disorder is a real thing and we're not sure whether the community health workers are equipped to be able to deal with that or even to see it within themselves." (I10)*

### NGOs: perspectives and roles in resilience building

#### The alignment of resilience with organisational priorities

As illustrated earlier in Table 1, each organisation has a distinct priority area of intervention and primary target groups, whether it be maternal and child health, HIV/AIDS, or people with disabilities. Nevertheless, over the years of implementation, the organisations realized the benefit of an overall health system strengthening approach to achieving their goals, which is why many of them have expanded their health programmes to take on a broader health system strengthening agenda. This meant an increased project investment towards training health workers, refurbishing key health infrastructure, and restocking supply of medicines as such.

*"it has to do with that we really expanded much more into health system strengthening that we have to look at it holistically from all sides. That was why it was a logical step to introduce this aspect of resilience and even to highlight it. We wanted to highlight it as a cross-cutting issue so that we actively work on it." (I06)*

In the same light, more NGOs started to view resilience as a cross-cutting issue. This has shifted their disease-specific perspective towards a more holistic, health system strengthening approach to programming, enabling multiple sectors working collaboratively to weave resilience into their respective programmes.

*"For us, but this is only recently with the new strategy since 2020, we look at resilience as a cross-cutting issue. We look at resilience when it comes to the engagements with the communities. But we haven't looked at it from a purely health system resilience aspect. That's also anyway a fairly new perspective on health system." (I06)*

Meanwhile, one NGO regarded resilience as an ultimate goal, even specified within the health results chain of its programmes with an underlying logic that “healthy people contribute to resilience.” This impact-driven approach to resilience brought about a fresh perspective that disaster risk management serves the purpose to build resilience for all.

*"The ultimate impact from the disaster risk management is that the resilience of the communities is improved, and the ultimate impact of our health result chain is that the health state of the people are improved. If you look at resilience and the resilience that we use as part of the resilience framework of the federation, is that healthy people contribute to resilience. So, actually the ultimate impact should be resilience for everybody." (I01)*

#### NGOs’ unique role for resilience building: knowledge exchange and science for impact

The NGOs believed that albeit their different views on resilience within their organisations, they nevertheless have a unique role in building resilience of their partner countries’ health systems thereby referring to the areas of knowledge exchange and innovations. Almost all participants believed that capacity building at both organisational and individual levels would be one of the best investments towards fostering resilience. There were already significant contributions from the NGOs through skills trainings of frontline workers and medical professionals. A participant believed that future investments should also prioritise in strengthening the capacity building of frontline workers and the communities in particular to equip resilience on the ground where the NGOs may draw on their strengths.

*"We still have some interesting things to bring is really the work at the community level and really building the capacities in terms of delivering first response at the community level, training community leaders and key community representatives with the basic package of first aid and mental health support. It doesn't necessarily need to be super technical. We can identify some people with basic backgrounds in social work or even first aid is accessible to anyone really. So that's really where we are trying to make a difference." (I20)*

One NGO pointed out that improving the capacity of the ministry of health itself and better coordinating with the ministry of education for licensing can also have an enormous effect in facilitating policy dialogue and enhancing the training quality of the entire system. The expected impact would be at a wider level as opposed to that from vertical programmes.

*"(what NGOs can do) Supporting people from far and really strengthening the capacity. I think NGOs can change enormously the way they work and absolutely collaborate with the ministry of health to improve the capacity. There are gaps everywhere, from medical point of view, psychological. I think NGOs should really specialise on these and supporting ministry of health rather than vertical programmes. And they could really reinforce the capacity of the central level and not only peripherical level. The NGOs should be involved in changing the current training and dialoguing with the ministry of health that the current initial training for health providers are improved." (I09)*

An additional role NGOs may take on is to facilitate dialogue and knowledge exchange not only from North to South, but also from South to South, where good practices can be mutually shared.

*This is what I see a bit as our role that when we have a good experience in Chad, we try to also make use of it and have this exchange (South South exchange), to facilitate the knowledge exchange." (I06)*

As most NGOs focused on a priority area of intervention, whether it be women and children’s health, gender violence, or HIV/AIDS, the NGOs were able to see issues from multiple angles, working with various ministries and partners to have their interventions benefit from a cross-sectoral approach. Resilience in most cases provided a link to connecting these different sectors.

*"Looking a bit beyond the health system. Women for example have been affected by fistula. They are not just physically affected but also psychologically and often they are isolated. Even if they are cured from the fistula, they still are super marginalised as they have been an outcast. So, we are also supporting with income generating activities to strengthen their opportunities with financial independence, which also in turn strengthen their resilience towards shocks in the future." (I06)*

Finally, the NGOs can support their partner countries to leverage science for impact. As the demand for adopting new digital tools and innovations is growing, one NGO interviewed has started introducing new mobile applications to improve children’s medical diagnosis and treatment, strengthening the overall primary health care system in rural West Africa [20]. In addition, innovative training modules have been adopted for health workers in order to adapt to the evolving context as well. This emerging need for digital approaches such as teleconsultations or telemedicine was most evident during the COVID-19 pandemic when travel restrictions compelled all exchanges to switch to virtual means. It will not work for all contexts, but the NGOs may be able to fund relevant technological and innovation gaps arising in their partner countries to maximise impact.

*"The entering of digitalisation to health workforce training, duel training models, how to use them for health workforce, that's for us the core element." (I08)*

### Future outlook: practical steps towards resilience building

#### Complex nature of future shocks

The shocks experienced by the low- and middle- income countries were no longer a disconnected, one-time event, but a series of complex, interconnected shocks that are increasing in both frequency and intensity. The nature of shocks were classified as political shocks and protracted conflicts, natural disasters and climate change, pandemics or epidemics, and financial crisis or economic shocks.

*"But the Sahel being so volatile with so many factors of stress and shock interconnecting, it's quite difficult to achieve impact..and most importantly for us, how it (conflicts) interacts with the prevalence of natural disasters in an area and just having exacerbate vulnerabilities where we work. So, the combination of these factors together..But at the same time, we have to acknowledge that this (epidemics/pandemics) is also a risk that is going to continue and it is linked with environmental degradation. We know that environmental degradation may lead to more health issues and epidemics." (I20)*

The most distressing aspect of all these shocks were that either multiple shocks were occurring in a country simultaneously or a shock has led to further shocks, aggravating contextual vulnerabilities and diminishing the impact of the NGOs’ interventions. For instance, a participant described how the financial crisis in Zimbabwe led to political and social unrests, which brought about a total shutdown of the health care system. Then the arrival of the COVID-19 pandemic and massive lockdowns thereafter, further depreciated the value of the currency. In many contexts, the root causes of the shocks were often interconnected and the NGOs found this a severe bottleneck to achieving impact. Hence, it appeared necessary for the NGOs to take a holistic approach beyond the health sector to understanding the shocks and stresses in the system in order to better interrogate resilience.

*"It was a whole cascade of things. There was the currency collapsing, political distress and strikes of course, people who got very upset … That (economic crash) was 2019 and then corona came. It was really bad. You had a very fragile system, and then you had the economic collapse, then you had strikes, so literally there were no people in the hospitals anymore, in the pharmacies. If people had problems, public health was not existing anymore. Then came corona and massive lockdowns, the value of the money was down." (I11)*

#### Strongest determinant of resilience: investing in context-based long-term health system hardware and software

According to the participants, the strongest determinant of resilience in the health system was the degree of investments made for building long-term health system infrastructures. Health systems are comprised of *hardware* and *software* components [21]. *Hardware* such as physical buildings, equipment, ambulances were equally critical as system *software* such as strong capacity of human resources and a shared work ethic. One respondent emphasized that the combination of these hardware and software ought to be sufficiently established and functioning prior to any potential crises.

*"(the most important element in health system resilience is) to have sufficient supplies, to have stocks, to have sufficient human resources, to have an infrastructure that withstands disasters that's non-existing. So, what resilience are we talking about if even the basics are not there. That's where I personally feel that the issue should be to work and contribute towards a resilient health system. Maybe first of all a functional and after the functional, resilient, as that is not guaranteed in all countries." (I01)*

Many organisations saw that rather than having a specific disaster preparedness plan, an overall strengthening of the health system would better contribute to building systemic resilience. For instance, when there are committed and competent nurses in the field, they would be the ones administrating COVID-19 vaccinations and serve in the frontlines during the crisis.

*"to make it more resilient, I think it is less important to have a specific disaster preparedness, but rather to strengthen the health system as such. Is there much more nurses on the spot, more midwives on the spot, these people stay on the spot also in a flooding or in an Ebola crisis, or in a COVID crisis. If you have a health workforce, they can do a COVAX vaccination now. If you don't have them, you can't. It's not about once you have a crisis, then you need to react. You need to really strengthen the system before. Indeed a system with a sufficient number of infrastructure, equipment, medication, well-trained health workers, and community which is interacting well, is quite resilient." (I08)*

When approaching resilience building, it is crucial to understand and build on the unique contextual strengths of the country’s health system. Whether it be through existing community health worker structures, doctor brigades, or traditional birth attendants, countries themselves should leverage their health care system’s assets to see what works best in their given context.

*"Why not distribute it through the house doctors when this is our network distribution? It's a matter of knowing your health system well enough. I think one important thing for resilience is to build on the strength of the system that you have. Know the strength, build on them." (I01)*

*"For example, we work with traditional birth attendants. They are not considered part of the health system in many countries but still they are the closest to the patients and therefore it's good to work with them and to make their services better. So, it's not an either or. You have to look at the picture and see where it makes more sense in the moment in time to strengthen which system." (I06)*

A case in point comes from Cuba where the government had invested in building a family doctor system, allocating a doctor and a nurse to serve each catchment area. During the COVID-19 pandemic, this system was fully utilised to enable a door-to-door service to monitor the health of its people and conduct contact tracing.

*"For example, one of the ways to prevent COVID was to look closely to the person that has symptoms or there were contacts. They used family doctors and medical students going from door to door everyday to check the temperature of the people. 20,000 medical students. Because of this, the first two waves of COVID in 2020 were very well controlled by the government because of this. The contact cases were alerted and ones that were vulnerable or had health problems they went to the hospitals to follow up. At the end of 2020 there were only a little more than 100 deaths in the whole Cuba." (I14)*

## Discussion

This article illuminates insights from Swiss NGOs dealing with unexpected shocks in the health systems in their partner countries. The key findings were summarised into the roles of the governments and the NGOs in resilience building, whilst acknowledging the unique contextual factors that ought to be considered in the process. The following four key ‘foundations of resilience’ were found to be dominant for unleashing resilience attributes: ‘realigned relationships,’ ‘foresight,’ ‘motivation,’ and ‘emergency preparedness.’ The attribute to ‘integrate’ was shown to be one of the most crucial characteristics of resilience expected of the national governments from the NGOs, which points to the heightened role of governance and the government’s responsibility as the primary duty bearer. The NGOs saw themselves in a unique position to target their investments towards facilitating knowledge exchange and supporting long-term adapations of innovative solutions that are increasingly demanded in their partner countries. The investments from NGOs are not merely monetary but rather an opportunity to seek innovative solutions to problems and to keep diverse players in global health accountable. Finally, context-based long-term health infrastructure surfaced as the strongest determinant of resilience, which would serve as entry points for future investments.

This research also offers a timely response to the recent call for a shift of resilience research from theory to practice by employing a bottom-up approach to applied research [22]. The experiences and perspectives of the NGOs reflected here echo the standpoints of practitioners and communities they represent on the ground, and add a multidisciplinary lens to understanding resilience. Furthermore, the role of non-state actors, namely the NGOs have become inevitably more powerful not only due to its large and diversified funding channels, but also due to its growing governance capacity in applying pressure to conventional leaders within global health governance to provide necessary checks and balances. The Ebola epidemic in 2014 has revealed that Médecins Sans Frontières, an NGO, can assume a leading role to jump start relief efforts, to apply pressure on the WHO to accelerate support and to assist governments’ compliance with the International Health Regulations (IHR) [23]. The following observations draw on the insights on the role of different stakeholders and their respective contributions towards resilience building.

First, the underlying view of resilience as a cross-cutting issue for many NGOs enables a holistic approach beyond the health sector for its interrogation. This study found that the nature of shocks in the health system are often complex and its root causes interconnected between multiple societal disciplines. For example, the financial crisis in Zimbabwe led to political and social unrests, which paralysed the health care system. The advent of the COVID-19 pandemic and lockdown measures have further depreciated the currency, leading to a vicious cycle. Hence, the interviewees conceded that a health system strengthening approach have typically helped reach their programme goals more effectively than focusing solely on their distinct priority area of interests, whether it be maternal child health, HIV/AIDS, or people with disabilities. Furthermore, placing resilience within the NGOs’ strategy have altered their disease-specific perspectives and provided a link for different sectors and disciplines to work collaboratively together.

Drawing from the social network analysis, the value of interdependence is described by Blanchet at al as the capacity of resilience to engage with a diverse group of actors belonging to a wider socio-political structure [7]. A platform of cross-sectoral actors working harmoniously at the sub-national level has for example been observed in Kenya where health facility staff, sub-county managers and local public administrators had worked in conjunction to pacify political tension [10]. Gilson et al. define this process as ‘collective sensemaking,’ an approach to organise different actors in achieving collective health goals based on power sharing and trust building. ‘Collective sensemaking’ has been manifested in the process of the NGOs shifting their disease-specific lens towards a holistic health system perspective in managing their programmes and weaving resilience into their respective strategies to better prepare for future crises. Haldane echoes the power of this holistic approach to resilience by stressing that in the management of the COVID-19 pandemic, the high-performing countries adopted a comprehensive response with multi-ministry task forces to draw upon capacities within and beyond the health system [24]. South Korea’s approach to the pandemic control has also been exemplified by a synergy of collaborative governance, confiding in the civil society and the public for its effectiveness [25]. Resilience building is best approached from a collaborative angle involving diverse actors.

Second, the role of robust governance was a feature of resilience expected of national governments by the majority of the interviewees reflecting Swiss NGOs. The European Observatory on Health Systems and Policies released a report summarising the key lessons generated from the COVID-19 pandemic. One recommendation was to improve health governance at the global level through creating a Global Health Board under the auspices of the G20 and to draft a Pandemic Treaty that holds governments accountable to those in need [26]. From our interviews with the NGO representatives, however, it was apparent that the governments were ultimately seen as duty bearers, determining the effectiveness of the coordination of partners and the sustainability of contributions. Even International Health Regulations (IHR), which is regarded as one of the strongest tools to exert global health governance, is heavily dependent on national capacities and cooperation [27]. Many NGOs that participated in this study operated, however, in fragile, weak or failed states, and such fragility may undermine efforts to build resilience in the first place. Unlike in stable contexts where states and citizens negotiate societal needs and obligations, fragile states lack this political process to reconcile the state-society expectations and to establish legitimacy [12]. In the context of fragility, our study participants observed that strong community structures filled vacuums created by the lack of state governance and facilitated resilience through the solidarity of the participating communities. This expanded concept of governance in the health system includes a range of governance agents including patients and communities as well as sub-national actors influenced by formal and informal rules shaping their dynamic [28]. Participatory political processes reinforce resilience of the state by offering collectively agreed strategies to deal with shocks [12]. Furthermore, communities that cooperate for a common good to exercise their civic duty, which is an indication of a higher civic capital, are proven to be more resilient to crises and recover faster than their fragmented counterparts [29]. Hence, the everyday practice of governance may be equally influential in the achievement of equity and thereafter resilience [30].

Third, as the NGOs pointed out, they can leverage their global and local presence to foster a bi-directional knowledge exchange and to support their partner countries utilise science for impact. Well-adapted digital innovations can support the flexibility required in times of crises. During the COVID-19 pandemic, the countries that have been pre-equipped with digital innovations such as telemedicine have reaped its benefits particularly during the restrictions of the pandemic [31]. We see evidence of contextualised digital solution transforming the way health workers in rural areas diagnose and treat children with increased accuracy and greater impact [20]. There has also been numerous small-scale cost-effective innovations that have been initiated at community and health service and hospital level [32]. Alam et al. rightly point out that a bi-directional exchange of innovations between the global South and North will contribute to resilience building in the long run [33]. NGOs are in the position to facilitate this knowledge exchange and to fund relevant technological gaps by targeting and redirecting their investments. A caveat here is not to be complacent with short-term adaptations that simply add responsibilities to the individuals in the frontlines, but to focus on long-term adaptations of innovations through an incremental process of continuous organisational learning, knowledge exchange, and trust building where the impact can be sustained [34].

Finally, the strongest determinant for resilience identified by the NGOs were health system hardware and software that require long term investments [21]. The findings were consistent with that of other resilient research, revisiting the importance of investing in the ‘slow variables’ such as building a sustainable career path of essential health workers and improving the structural capacity of its health infrastructure catering to its context-specific risk [6, 11, 35]. Barasa et al., however, point out that effective planning processes, management capacities, and productive work cultures, all representing system software components, may matter more to resilience than adequately resourced hardware of the system. A strong software can effectively navigate through its complex power dynamics to influence action for strengthening the other parts of the health system [9]. A purposeful designing and redesigning of the country’s institutions in anticipation of future shocks will prevent the system from ‘coping’ [36] or developing ‘maladaptive’ practices [37] in response to acute stressors and shocks. True resilience reflects an ongoing journey of the country’s institutions and participating actors to gradually process through its absorptive, adaptive, and transformative capacities [38], leveraging its unique contextual strengths and offsetting its health system vulnerabilities.

### Study strengths and limitations

As an applied research, this study builds on a health system resilience conceptual framework developed from a previous systematic review and complementary research on Myanmar [11, 39]. This article presents empirical evidence on health system resilience through analysing the perspectives of NGOs operating in low- and middle- income countries. The topic of health system resilience has a potential to make a timely contribution to the health systems and policy research community, with lessons generated that can be transferrable to all contexts that operate in close partnerships between the global South and North. Due to the COVID-19 pandemic’s travel restrictions imposed during the data collection period, the study only represents the view of Swiss-based NGOs, restricting the scope of the study to the perceptions of a single stakeholder group. As the interviewees were selected using a snowball sampling strategy through existing networks, this was applied through the Swiss network which the authors are a part of. In addition, it was also possible to virtually connect with many participants based in the field where projects took place. The heterogeneity of the NGOs’ size, primary target groups, and areas of interest, however, may pose limitations for a generalised application of the study. Yet, the diversity of the participants’ nationalities and previous work experiences have potentially added to the robustness of the findings. Further studies, however, should explore capturing a wider range of stakeholders, the perceptions of communities, frontline workers and government staff that may be able to offer a more holistic view of the topic.

## Conclusions

Health system resilience is a collective endeavour and a result of many stakeholders’ consistent and targeted investments. These investments open up new opportunities to seek innovative solutions and to keep diverse actors in global health accountable. The experiences and perspectives of the Swiss NGOs in this article highlight the vital role NGOs may play in building resilient health systems in their partner countries. Specifically, strong governance, a bi-directional knowledge exchange, and the focus on leveraging science for impact can draw greater potential of resilience in the health systems. Governments and the NGOs have unique points of contribution in this journey towards resilience and bear the responsibility to support governments to prioritise investing in the key ‘foundations of resilience’ in order to activate greater attributes of resilience. Resilience building will not only prepare countries for future shocks but bridge the disparate health and development agenda in order to better address the nexus between humanitarian aid and development cooperation.

## Supplementary Information

**Additional file 1.**

## Data Availability

The dataset presented in this article are not publicly available. Making the full data set publicly available could potentially breach the privacy that was promised to participants when they agreed to take part and the ethics approval granted from the Ethikkommission Nord-west und Zentralschweiz in Switzerland. The interview guide which contains the list of questionnaires is provided as a supplementary file. Requests for access to the full dataset can be directed to the corresponding author.
