# Supplementary material for: What makes health systems resilient? A qualitative analysis of the perspectives of Swiss NGOs
Source: Global Health. 2022 May 26;18:55. doi: 10.1186/s12992-022-00848-y (PMC9134130; doi:10.1186/s12992-022-00848-y)
Supplement: Supplementary file 1 — Additional file 1. [file 12992_2022_848_MOESM1_ESM.pdf]

Supplementary File 1. Interview Guide

Study Title: Health System Resilience: Perspectives of Swiss-based NGOs

Investigator: ________________________ **Phone number:**___________________________
Organisation/Department: ______________________________ **Date:** |__|__|__|__|__|__|
Position/Title: ____

IN-DEPTH INTERVIEW GUIDE

Introduction: purpose of research, use of audio tape recorder, confidentiality

I. PARTICIPANT INTRODUCTION

1. Can you please describe your organisation's engagement with health systems in low- and middle-income countries?

2. What is your role when there is an external shock, whether it be a natural disaster, an unforeseen pandemic, political crises, etc. occurring in the countries your organisation is partnering with?

Prompt: examples of disaster management, pandemic, natural disasters

II. EXPERIENCE OF HEALTH SYSTEM SHOCKS

3. Can you please describe in detail what kinds of shocks on health systems your organisation have experienced through the countries you have worked with in the last 10 years?

Probe: What was your title/position at the time?
What was the response protocol (written or perceived norm)?
What actions (out of the norm) did you take?

4. What kinds of support did your organisation provide in the event of such shocks?

Probe: Decision-making protocol? Evidence? Reports?

5. What key problems have you encountered during this process and what were the reasons for these challenges?

6. What actions were taken to overcome problems and what has or hasn't worked?

7. What were the main lessons learnt after undergoing this experience?

III. DIMENSIONS OF HEALTH SYSTEM RESILIENCE

7. How would you define health system resilience based on your organisation's experience?

Probe: Definition of health system resilience?

8. What kind of structure (e.g. framework, policy, protocol, guidance) if any, does your organisation rely on when dealing with a shock in your partner countries?

9. Which components do you think are critical for resilience when dealing with your partner countries' shocks?

10. What factors facilitate or hinder a resilient health system?

IV. SELF-ASSESSMENT

11. How would you assess the level of support your organisation makes towards building the resilience of health systems in your partner countries?

12. Do you have a monitoring mechanism in place to track progress in this aspect?

V. PREPAREDNESS AND FUTURE SHOCKS

13. What may be the biggest threat/risk for your partner countries?

14. At this point in time, how would you describe your partner countries' preparedness for future shocks?

15. What would you advise to improve health system resilience in your partner countries?

Thank you for your time and heartfelt responses to the interview questions.
